# Supplementary figures and images for: Multi-year pair-bonding in Murray cod (Maccullochella peelii)
Source: PeerJ. 2020 Dec 10;8:e10460. doi: 10.7717/peerj.10460 (PMC7733648; doi:10.7717/peerj.10460)

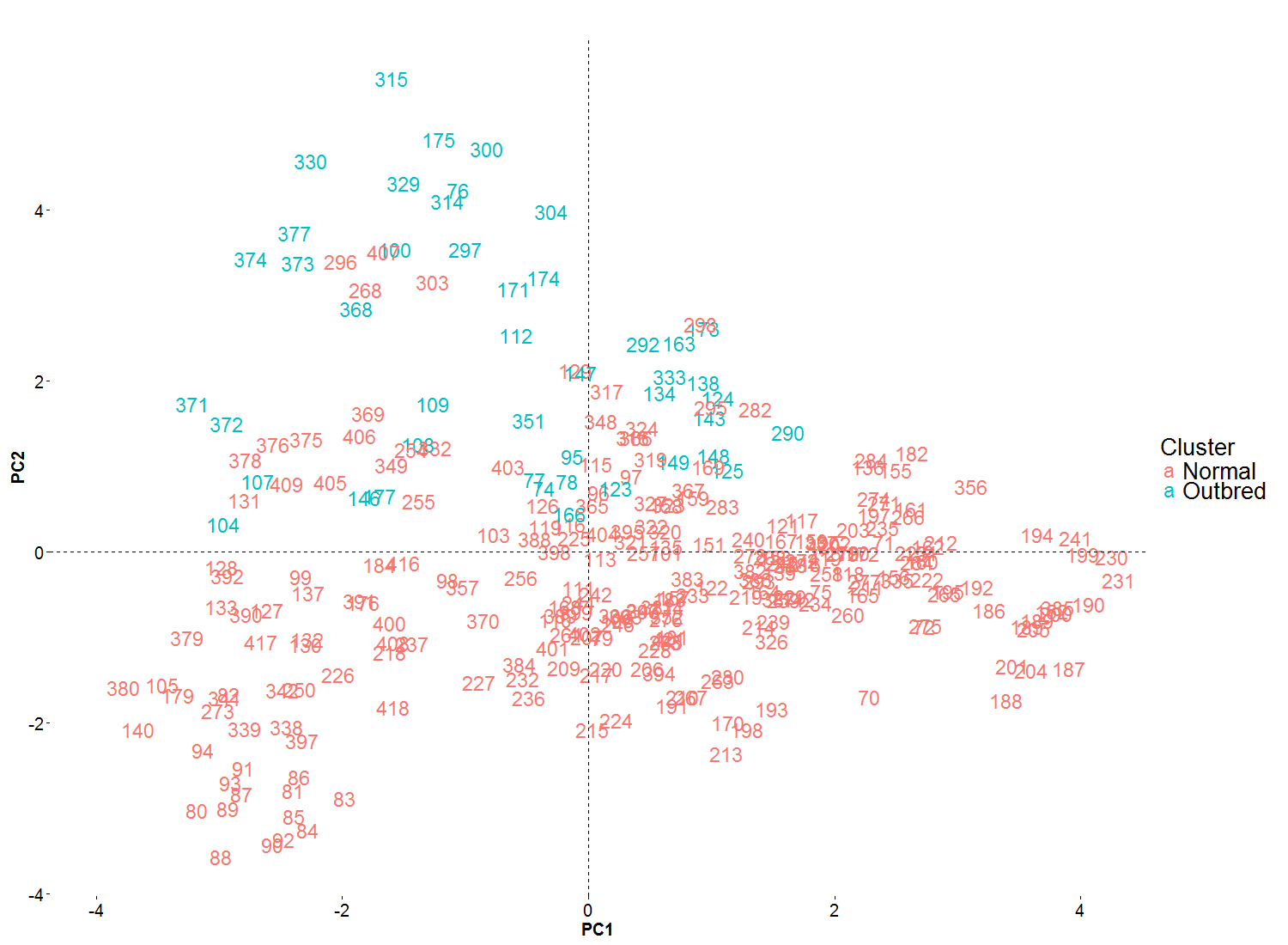

Supplement: Supplemental Information 1 [file peerj-08-10460-s001.png]
